# Islet‐specific CD8 ^+^ T cells gain effector function in the gut lymphoid tissues *via* bystander activation not molecular mimicry

**DOI:** 10.1111/imcb.12593

**Published:** 2022-11-01

**Authors:** Mirei Okada, Vivian Zhang, Jeniffer D Loaiza Naranjo, Bree J Tillett, F Susan Wong, Raymond J Steptoe, Anne‐Sophie Bergot, Emma E Hamilton‐Williams

**Affiliations:** ^1^ The University of Queensland Diamantina Institute, The University of Queensland Woolloongabba QLD Australia; ^2^ Division of Infection and Immunity and Systems Immunity University Research Institute, School of Medicine Cardiff University Cardiff UK; ^3^ Present address: The Charles Perkins Centre and School of Life and Environmental Sciences University of Sydney Camperdown NSW 2006 Australia

**Keywords:** autoimmunity, bystander activation, islet‐specific T cells, microbiota, molecular mimicry, Type 1 diabetes

## Abstract

Type 1 diabetes (T1D) is caused by aberrant activation of autoreactive T cells specific for the islet beta cells. How islet‐specific T cells evade tolerance to become effector T cells is unknown, but it is believed that an altered gut microbiota plays a role. Possible mechanisms include bystander activation of autoreactive T cells in the gut or “molecular mimicry” from cross‐reactivity between gut microbiota‐derived peptides and islet‐derived epitopes. To investigate these mechanisms, we use two islet‐specific CD8^+^ T cell clones and the non‐obese diabetic mouse model of type 1 diabetes. Both insulin‐specific G9C8 cells and IGRP‐specific 8.3 cells underwent early activation and proliferation in the pancreatic draining lymph nodes but not in the Peyer's patches or mesenteric lymph nodes. Mutation of the endogenous epitope for G9C8 cells abolished their CD69 upregulation and proliferation, ruling out G9C8 cell activation by a gut microbiota derived peptide and molecular mimicry. However, previously activated islet‐specific effector memory cells but not naïve cells migrated into the Peyer's patches where they increased their cytotoxic function. Oral delivery of butyrate, a microbiota derived anti‐inflammatory metabolite, reduced IGRP‐specific cytotoxic function. Thus, while initial activation of islet‐specific CD8^+^ T cells occurred in the pancreatic lymph nodes, activated cells trafficked through the gut lymphoid tissues where they gained additional effector function *via* non‐specific bystander activation influenced by the gut microbiota.

## Type 1 diabetes is caused by aberrant activation of autoreactive T cells specific for the islet beta cells, but how these cells evade tolerance to become effector T cells is unknown. This study investigated whether islet‐specific T cells are activated in the gut *via* cross‐reactive bacterial derived peptides (molecular mimicry) or by non‐specific bystander activation. While initial activation of islet‐specific CD8^+^ T cells only occurred in the pancreatic lymph nodes, activated cells migrated to the Peyer's patches where they gained additional effector function under the influence of the gut microbiota.

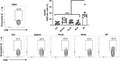

## INTRODUCTION

Many autoimmune diseases such as type 1 diabetes (T1D) have been increasing steadily in incidence since the mid‐20th century. T1D is caused by a T‐cell‐mediated attack on the insulin‐producing beta cells in the pancreas. The increase in disease is attributed to environmental changes that impact the activation of the autoreactive T‐cell response.^1^ One such environmental factor with increasing evidence for a role in the pathogenesis of T1D is a change in the colonization of gut bacteria known as the microbiota.^2^ In this scenario, a more inflammatory gut bacteria population is thought to colonize the gut, either due to altered diet, lifestyle or perturbations caused by antibiotics or other environmental influences. This altered bacterial population can influence T‐cell activation by producing altered endotoxins such as lipopolysaccharide (LPS),^3^ reducing the integrity of the gut barrier allowing the passage of inflammatory signals,^4^ reducing the production of anti‐inflammatory metabolites such as short‐chain fatty acids^5^ or *via* direct activation of islet‐specific T cells through molecular mimicry.^6^

Molecular mimicry is a mechanism whereby cross‐reactivity occurs between T cell‐targeted, beta cell‐derived epitopes in the pancreas and exogenous viral or bacterial derived peptides. A T cell can then become activated and mature into an effector cell by the bacterial or viral derived mimic peptides in an inflammatory context, and then migrate to the pancreas and attack the beta cells. In non‐obese diabetic (NOD) mice, a high proportion of the CD8^+^ T‐cell compartment invading the pancreatic islets close to the time of disease onset has been shown to recognize islet derived antigen Islet‐specific glucose‐6‐phosphatase catalytic subunit‐related protein (IGRP) amino acids 206–214.^7^ Human islet‐specific T cells also target IGRP‐derived peptides.^8^, ^9^ Cross‐reactivity of IGRP_206‐214_ CD8^+^ T cells has been demonstrated for a mimic peptide expressed by a Fusobacteria phylum member, *Leptotrichia goodfellowii*, found in both the human and NOD mouse commensal population.^6^ In another study, IGRP_206‐214_ specific T cells were shown to recognize and to be activated by an integrase protein encoded by several *Bacteroides* genus members including *B. vulgatus* and *B. dorei*.^10^ Bacteroides genus members including *B. vulgatus* and *B. dorei* have been shown to be increased in the gut microbiota of individuals with or at‐risk of T1D.^2^, ^3^, ^11^, ^12^, ^13^

T cells specific for various epitopes within insulin and proinsulin are believed to be critical for progression of T1D in both humans and the NOD mouse. Insulin B chain amino acids 9–23 (InsB_9‐23_; a CD4^+^ T‐cell epitope), and the overlapping CD8^+^ T‐cell epitope InsB_15‐23_ are thought to be key disease initiating epitopes.^14^, ^15^, ^16^, ^17^ InsB_15–23_ has been shown to be amongst the earliest detected epitopes targeted by CD8^+^ T cells in young NOD mice,^18^ and is very similar to the human InsB_15‐24_ epitope targeted by islet‐reactive CD8^+^ T cells in diabetes‐susceptible HLA‐A24*02 positive individuals.^19^ Evidence for bacterial mimics for insulin epitopes including insulin_B9‐23_ has been reported, although it has not been directly shown whether the bacterial derived peptides can activate insulin‐specific T cells *in vivo*.^20^, ^21^ Furthermore, T‐cell receptor (TCR) cross‐reactive between islet and gut commensal epitopes have been discovered in human type 1 diabetes, but their role in disease pathogenesis is unknown.^22^, ^23^ In NOD mice, it was shown that altering the gut microbiota composition *via* treatment with antibiotics influenced the activation and expansion of InsB_15‐23_ specific CD8^+^ T cells.^24^, ^25^ However, a direct assessment as to whether molecular mimicry *versus* bystander activation plays a role *in vivo* in priming the initial islet‐specific T‐cell responses is missing.

Here, we investigate whether early activation of CD8^+^ T cells specific for IGRP_206–214_ or InsB_15–23_ occurs in the pancreatic‐draining lymph nodes (PcLN) alone or also occurs in the gut‐associated lymphoid tissues (GALT). We used NOD mice with a mutated proinsulin transgene with an alanine at position B16, which abolishes binding of the InsB_15–23_ peptide to the H‐2K^d^ complex together with a knockout of both native insulin genes to investigate the dependency of InsB_15‐23_ specific T cells on pancreatic rather than microbiota‐derived antigen for their activation.^14^, ^26^, ^27^, ^28^ While initial activation of islet‐specific CD8^+^ T cells only occurred in the PcLN, activated cells migrated to the Peyer's patches (PP) where they gained additional effector function under the influence of the gut microbiota.

## RESULTS

### Early activation of both 8.3 and G9C8 transgenic T cells occurs in the PcLN but not the PP or mesenteric lymph nodes

**Figure 1:**
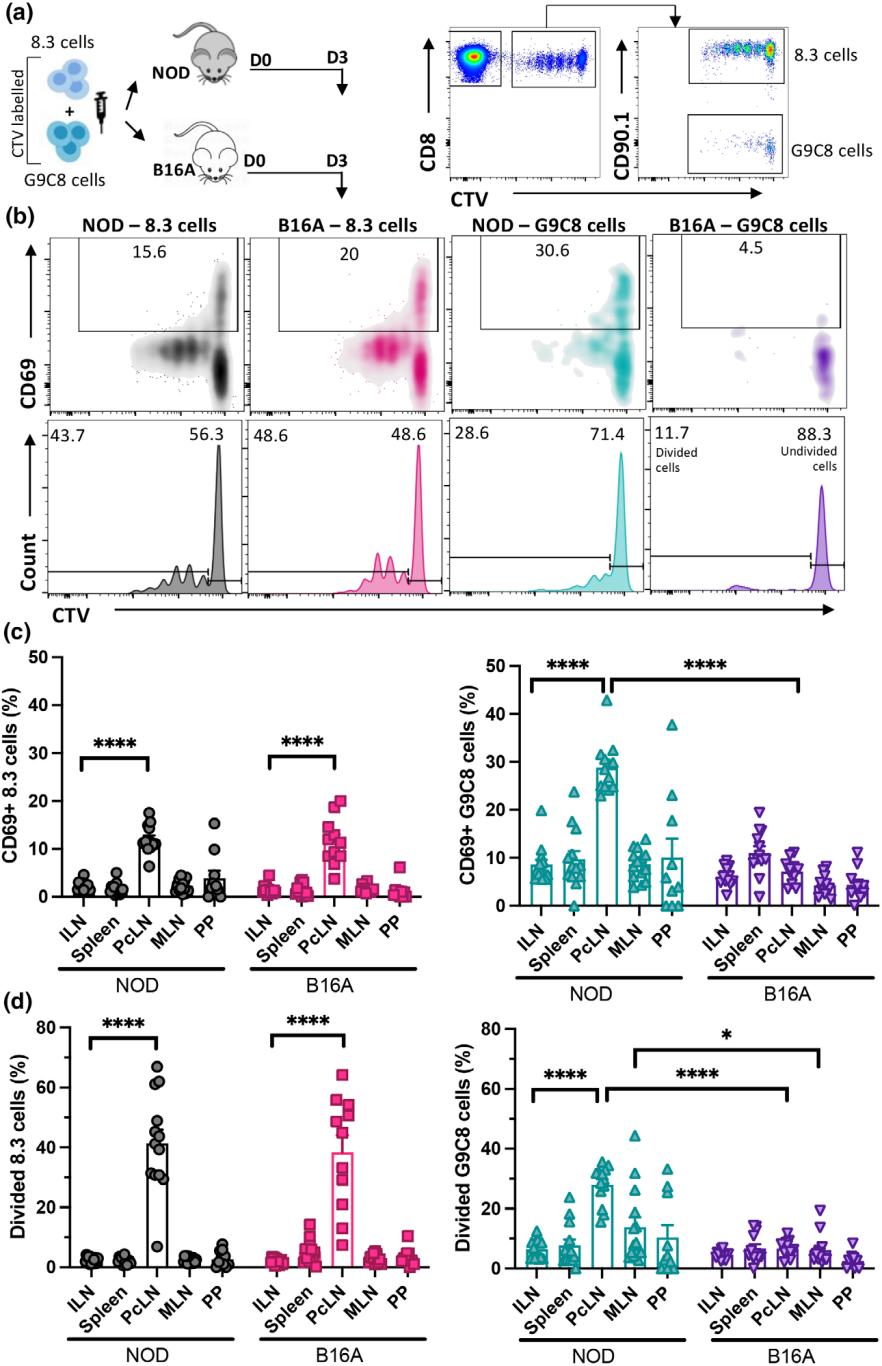
8.3 and G9C8 cells are activated and proliferate in the PcLN of NOD mice, but G9C8 cells fail to be activated in B16A recipients. **(a)** Experimental design. Transgenic CD90.1^+^ 8.3 cells and CD90.1^−^ G9C8 cells were CTV labeled, mixed and injected into NOD and B16A recipient mice. Samples were analyzed at day 3. Donor cells were identified using CTV and CD90.1 expression. **(b)** Representative plots of CD69 (top) and CTV (bottom) in 8.3 cells in PcLN from NOD (gray) and B16A (pink) recipients; G9C8 donor cells from NOD (green) and B16A (purple) recipients. **(c)** Proportions of CD69 and **(d)** divided 8.3 cells and G9C8 cells. Data were pooled from three different experiments (*n* = 14 total with 4 or 5 mice per experiment). Mean ± SEM is shown for each group, statistical analysis was performed using two‐way ANOVA with Tukey's multiple comparison test. *P*‐value * < 0.05, **** < 0.0001.

We investigated whether IGRP_206‐214_ specific transgenic 8.3 CD8^+^ T cells and InsB_15‐23_ specific transgenic G9C8 T cells are first activated only in the PcLN or also in the small intestine PP or in the mesenteric lymph nodes (MLN) which drain the large intestine. Skin‐draining inguinal lymph nodes (ILN) were included as a control where we did not expect to observe any activation of islet‐specific T cells. Transgenic 8.3 cells (CD90.1^+^) and G9C8 cells (CD90.1^−^) were mixed at a 1:1 ratio, labeled with cell‐tracking dye Cell‐Trace Violet (CTV) and adoptively transferred into NOD mice (Figure 1a). Three days after transfer, the expression of the early activation marker CD69 significantly increased on both 8.3 and G9C8 cells in the PcLN but not in the ILN, spleen, MLN or PP (Figure 1b, c). Although there were individual outlier mice with CD69 expression on the transferred CD8^+^ T cells in the PP as high as in the PcLN, this was not significant. Similarly, CTV low, divided 8.3 cells were only observed in the PcLN but not in other tissues (Figure 1b, d). G9C8 cells tended to divide to a lower overall extent than 8.3 cells and were only found to a significant degree in the PcLN, although again some outlier mice had dividing G9C8 cells in the MLN and PP (Figure 1d). We concluded that the primary site of antigen‐experience for both 8.3 and G9C8 T cells is in the PcLN.

In order to definitively determine whether endogenous pancreas‐derived InsB_15‐23_ peptide alone drives activation of transgenic G9C8 cells, we also co‐transferred CTV labeled 8.3 and G9C8 cells into B16A NOD mice, which have been engineered to remove the native InsB_15‐23_ epitope by mutation of the B16 amino acid to alanine preventing the epitope binding to the H‐2K^d^ molecule.^14^, ^26^ Both upregulation of CD69 and proliferation of G9C8 cells in the PcLN was abolished in B16A NOD mice (Figure 1b–d). Interestingly, no “outlier” mice with higher CD69 or proliferating G9C8 cells were observed in the MLN or PP of B16A mice, and the proportion of divided G9C8 cells was significantly lower in the MLN of B16A mice compared with NOD mice. This suggested that these outlier cells in the NOD mice may have been derived from the migration of cells, which had first divided in the PcLN, into other tissues. As expected, activation of IGRP‐specific 8.3 cells was not impacted by the B16A proinsulin mutation and both CD69 upregulation and division of the transferred 8.3 cells occurred to a similar degree in the B16A and NOD mice (Figure 1c, d). These data provide evidence that early activation of G9C8 cells was dependent on the presence of the endogenous insulin epitope and not due to molecular mimicry of an exogenous bacterial peptide.

### Islet‐specific 8.3 and G9C8 cells with an effector‐memory phenotype are found in the PcLN and PP

**Figure 2:**
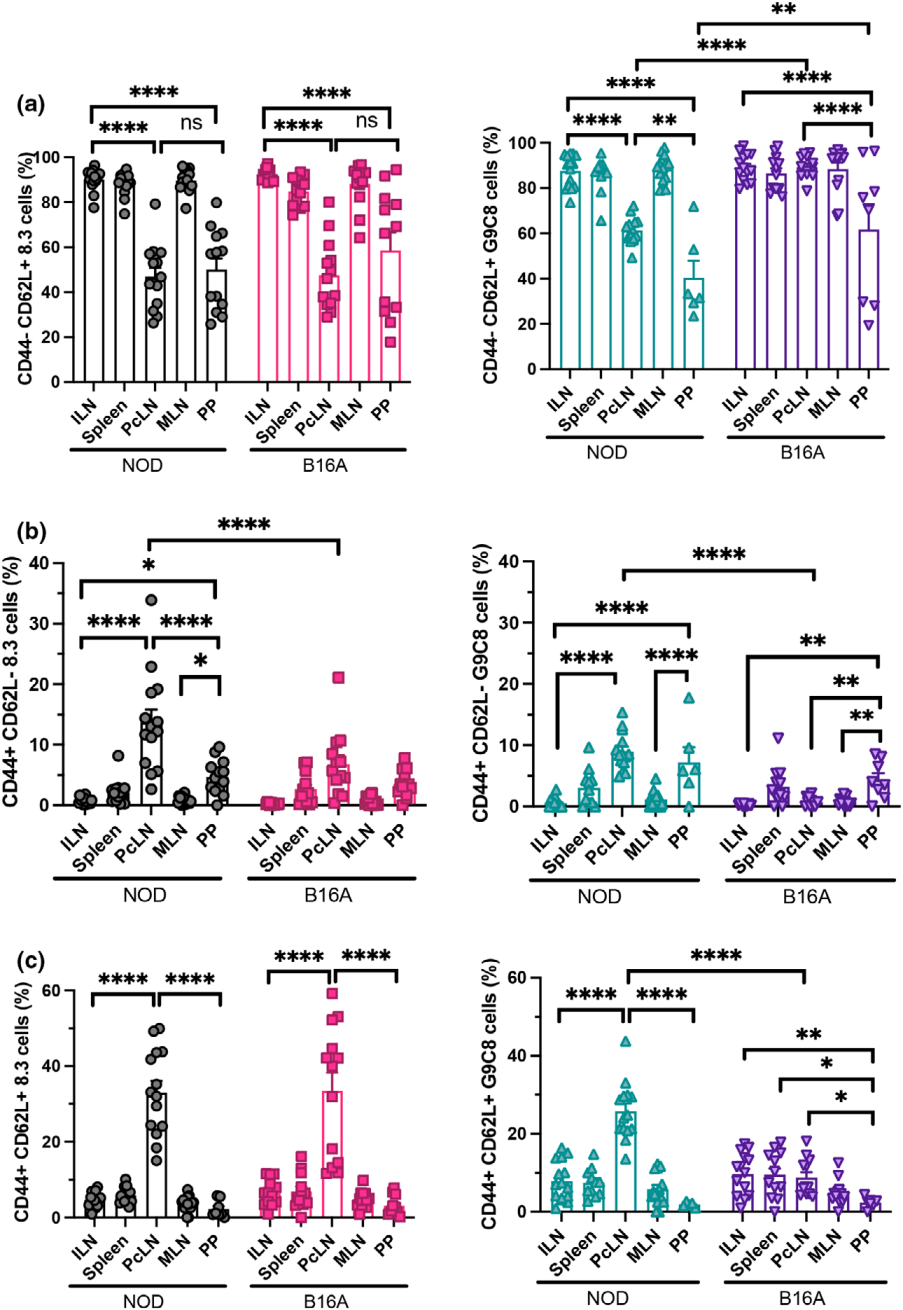
8.3 cells and G9C8 cells with an effector‐memory phenotype are found in PcLN and PP of NOD and B16A recipient mice. Transgenic CD90.1^+^ 8.3 cells and CD90.1^−^ G9C8 cells were injected into NOD and B16A recipient mice and analyzed at day 3. Proportions of naïve (CD44^−^CD62L^+^) **(a)**, effector memory (CD44^+^CD62L^−^) **(b)** and central memory phenotype (CD44^+^CD62L^+^) **(c)** in 8.3 cells from NOD (gray) and B16A (pink) recipients; G9C8 donor cells from NOD (green) and B16A (purple) recipients. Data were pooled from three different experiments (*n* = 14 total with 4 or 5 mice per experiment). Data are displayed as mean ± SEM. Statistical analysis was performed using two‐way ANOVA with Tukey's multiple comparison test. *P*‐value * < 0.05, ** < 0.01, **** < 0.0001.

Next, we phenotyped the activation status of the transferred 8.3 and G9C8 cells after transfer into the NOD and B16A mice. CD44 and CD62L markers were used to define naïve (CD44^−^CD62L^+^), effector memory (CD44^+^CD62L^−^) and central memory (CD44^+^CD62L^+^) populations within the transgenic cells (Supplementary figure 1). Surprisingly, although we had not found divided or CD69^+^ 8.3 or G9C8 T cells in the PP of NOD mice, we found a significant reduction in the proportion of naïve CD44^−^CD62L^+^ 8.3 and G9C8 T cells in both the PcLN and the PP. In contrast, the majority of transgenic cells in the ILN, MLN and spleen remained naïve (Figure 2a). This corresponded with an increase in effector memory (CD44^+^CD62L^−^) but not central memory (CD44^+^CD62L^+^) transgenic cells in the PP (Figure 2b), whereas central memory transgenic cells only accumulated in the PcLN (Figure 2c). Interestingly, in B16A mice, the reduction in naïve G9C8 cells and the increase in effector memory G9C8 cells in the PP still occurred, suggesting this was not dependent on the presence of the endogenous InsB_15–23_ epitope in the recipient mice. The proportion of effector memory 8.3 as well as G9C8 cells was decreased in the PcLN of B16A mice, suggesting that the generation of effector memory IGRP‐specific CD8^+^ T cells is dependent on the presence of the activated insulin‐specific T cells.

### Naïve 8.3 cells differentiate into effector cells in the PcLN, but not in the PP

**Figure 3:**
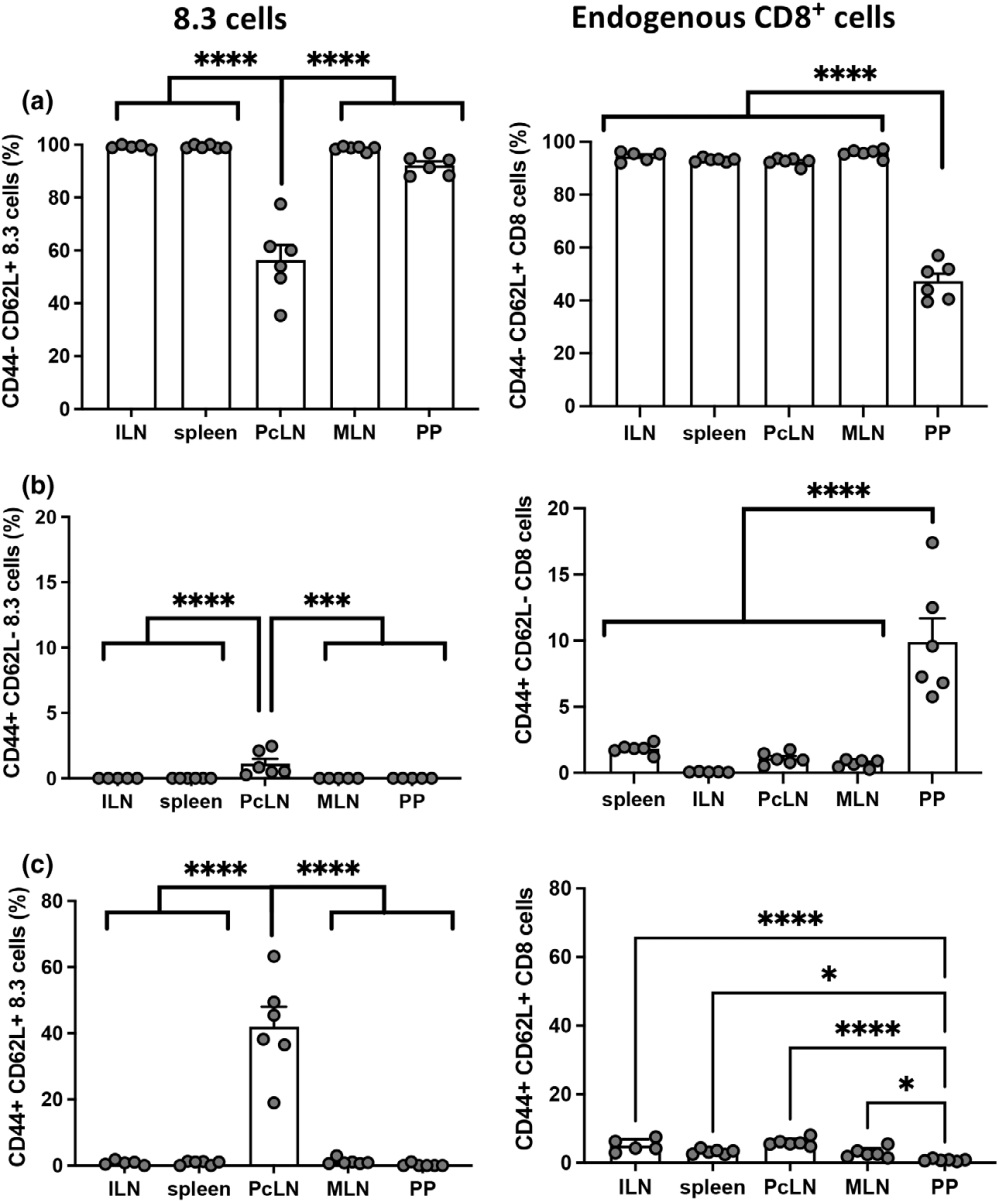
Naïve 8.3 cells differentiate into effector cells in the PcLN, but not in the PP. Purified CD8^+^ CD90.1^+^ transgenic 8.3 cells were sorted based on CD62L^+^ and CD44^low^ expression (naïve phenotype), transferred into NOD mice and analyzed at day 3. Proportions of naïve (CD44^−^CD62L^+^) **(a)**, effector memory (CD44^+^CD62L^−^) **(b)** and central memory phenotype (CD44^+^CD62L^+^) **(c)** in 8.3 CD90.1^+^ cells (left) or endogenous CD90.1^−^ cells (right). Data are representative of two independent experiments (*n* = 6 total and *n* = 3 mice per experiment). Data are displayed as mean ± SEM. Statistical analysis was performed using one‐way ANOVA with Tukey's multiple comparison test. *P*‐value * < 0.05, *** < 0.001, **** < 0.0001.

We hypothesized that the increase in activated islet‐specific T cells in the PP was due to the migration of cells previously activated by pancreatic peptide in the PcLN. This may have occurred in the B16A mice due to the presence of a small number of transferred cells that were already activated by islet antigen in the TCR transgenic donor mice. To test for this, we repeated the transfer of 8.3 cells into NOD mice using sorted CD44^−^CD62L^+^ naïve donor cells resulting in > 99% of donor 8.3 cells with a naïve phenotype compared with 90% naïve before sorting. When sorted naïve 8.3 cells were transferred into NOD mice, the decrease in naïve and increase in effector memory 8.3 cells was no longer observed in the PP (Figure 3a–c). However, within the endogenous CD8^+^ T‐cell population, the PP T cells had a low proportion of naïve T cells and increased effector memory T cells (Figure 3a–c). This confirmed that naïve 8.3 cells undergo their initial activation only in the PcLN where endogenous antigen is present and not in the PP where putative gut bacterial mimics peptide may be present.

### Endogenous IGRP‐specific CD8 ^+^ T cells within the PP are cytotoxic

**Figure 4:**
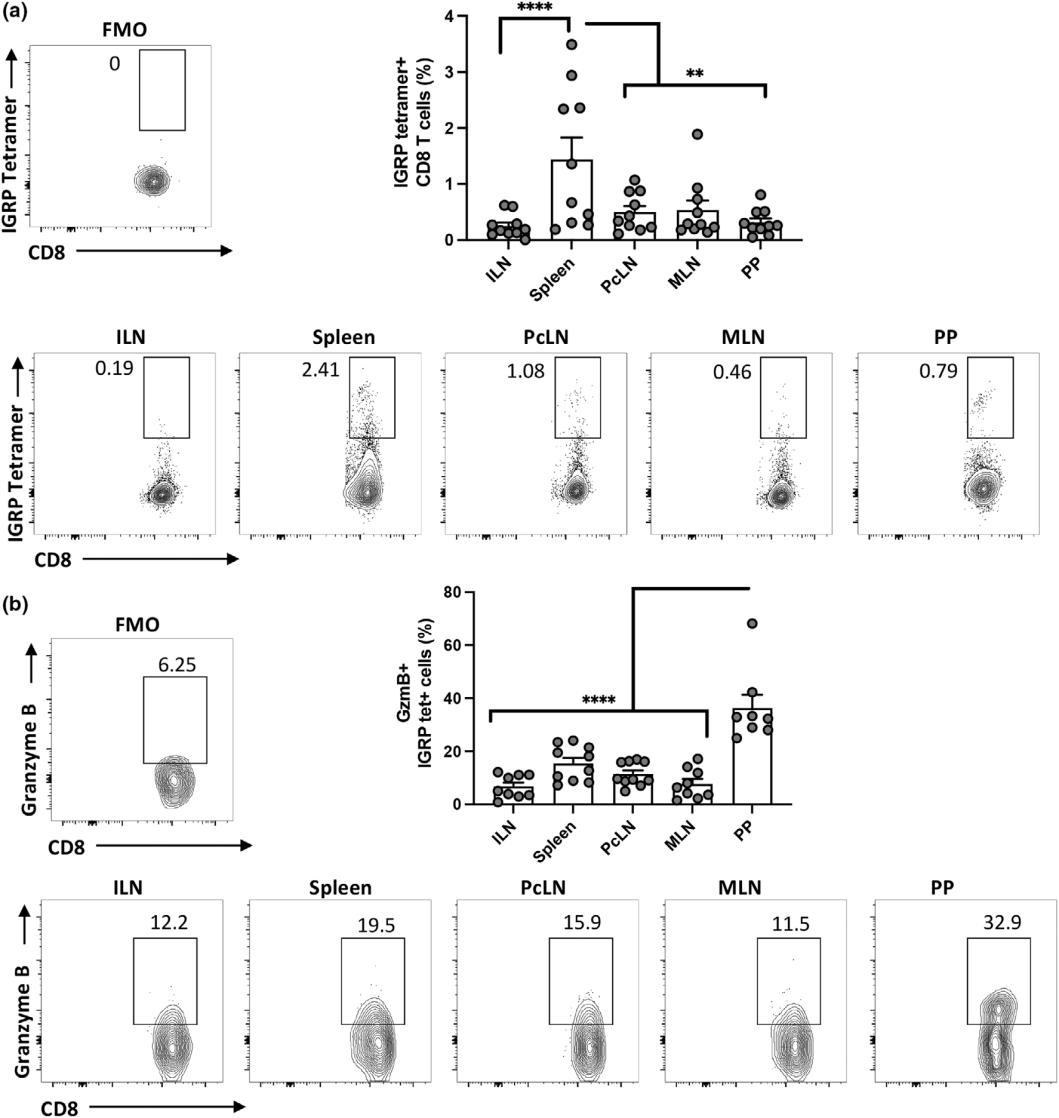
Endogenous IGRP‐specific‐specific cells express granzyme B in the PP of NOD mice. Tissues from 12–16‐week‐old female NOD mice were collected and stained with IGRP tetramer. **(a)** Representative contour plots of tetramer staining in CD8 cells, fluorescence‐minus‐one (FMO) control and summary graph. **(b)** Representative contour plots of granzyme B staining on IGRP tetramer^+^ cells and FMO control. Data were pooled from two different experiments (*n* = 10 mice total, *n* = 5 per experiment). Data are displayed as mean ± SEM, and statistical analysis was performed using one‐way ANOVA with Tukey's multiple comparison test. *P*‐value ** < 0.01, **** < 0.0001.

**Figure 5:**
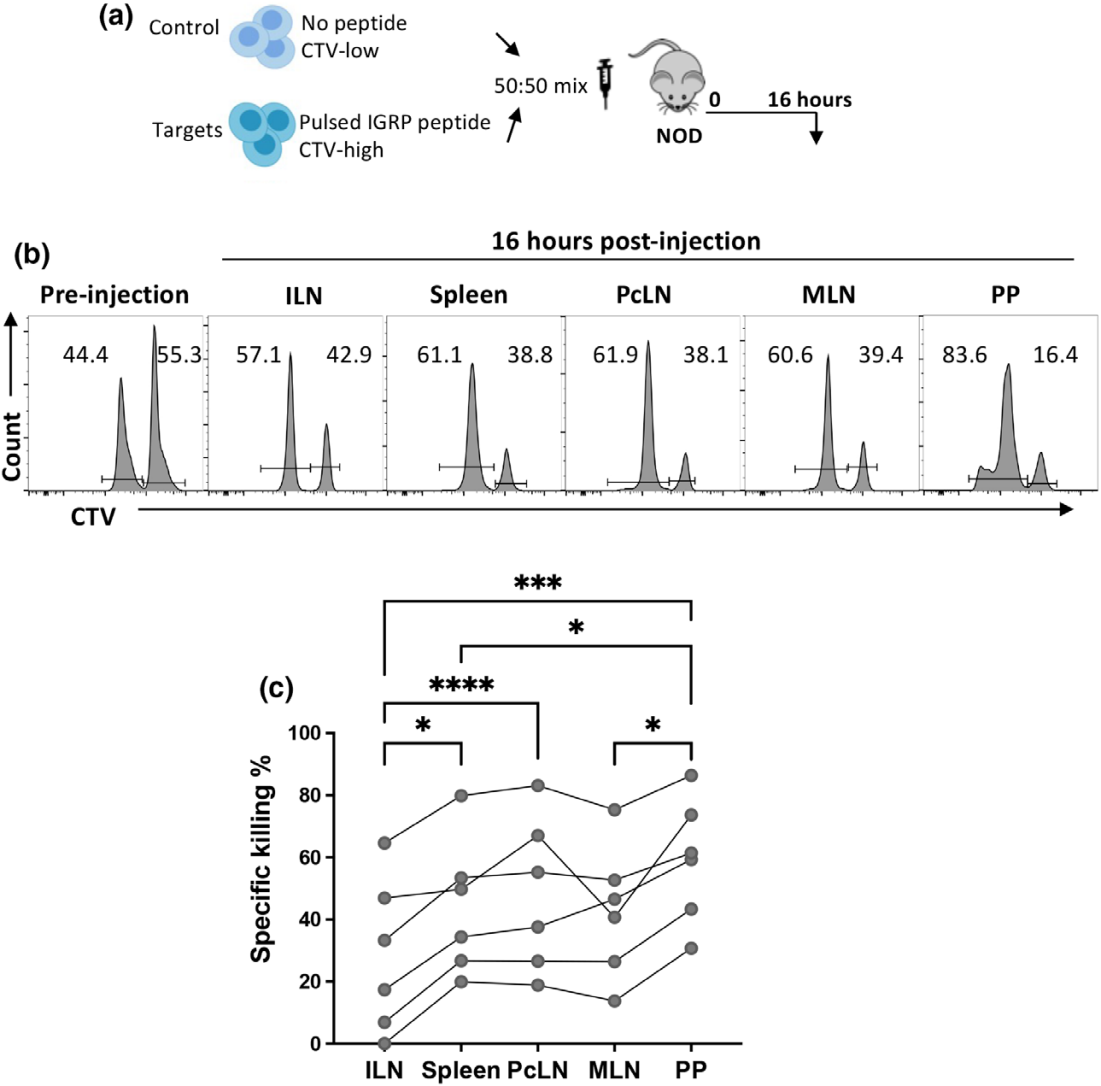
High IGRP‐specific cytotoxicity observed in the PP of NOD mice. **(a)** Experimental design. Untreated NOD mice received a mix of 5 million CTV^low^ control cells and 5 million CTV^high^ IGRP‐peptide‐pulsed target cells. Mice were killed after 15 hours, and tissues were collected for processing and analysis. **(b)** Representative histograms of CTV labeling in transferred cells pre‐injection (left) or in the respective tissues 16 hours later. **(c)** Specific killing on the different tissues. The experiment was performed twice (*n* = 6 per experiment). Data are displayed as mean ± SEM, statistical analysis we performed using one‐way repeated measures ANOVA with Tukey's multiple comparison test. *P*‐value * < 0.05, *** < 0.001, **** < 0.0001.

Next, we investigated effector function in endogenous islet‐specific T‐cells in the PP. IGRP_206–214_ specific CD8^+^ T cells were identified using tetramers in the various lymphoid tissues of NOD mice (Figure 4a). The proportion of the endogenous tetramer‐binding cells was not increased in the PP but tetramer^+^ cells were present. Granzyme B expression was increased in the PP on IGRP_206–214_ specific cells, suggesting the cells within the PP may be more cytotoxic compared with other tissues (Figure 4b). In order to directly test the cytotoxic capacity of IGRP_206‐214_ specific T cells in the various tissues, we performed an *in vivo* CTL assay as described previously.^29^ IGRP_206–214_ loaded target cells and control cells were CTV labeled and co‐transferred into NOD mice (Figure 5a, b). In line with the high expression of granzyme B in the PP, the highest IGRP_206–214_ specific killing was found in the PP and the lowest killing in the ILN (Figure 5c). Thus, islet‐specific T cells with a highly activated phenotype are found within the GALT.

### Delivery of bacterial metabolite butyrate reduces IGRP‐specific cytotoxicity

**Figure 6:**
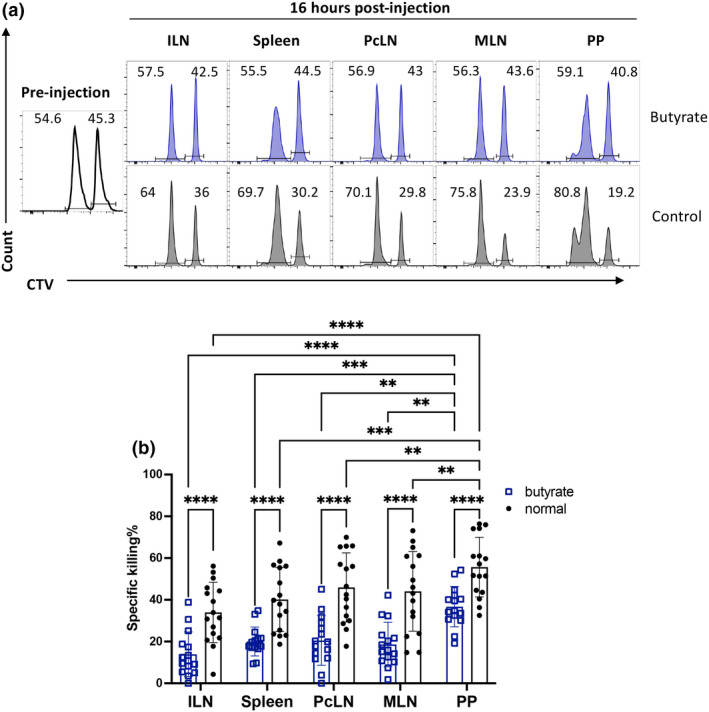
Oral butyrate reduces IGRP‐specific cytotoxicity in NOD mice. NOD mice were administered 200 mM butyrate in drinking water or normal water for 2 weeks prior injection of control and target cells. Mice received a mix of 5 million CTV^low^ control cells and 5 million CTV^high^ IGRP‐peptide‐pulsed target cells. Mice were killed after 16 hours, and tissues were collected for processing and analysis. **(a)** Representative histograms of CTV expression in transferred cells from mice that received butyrate (blue) or normal (gray) drinking water. **(b)** Specific killing. Data were pooled from two experiments (*n* = 16 mice total, *n* = 8 per experiment). Data are displayed as mean ± SEM, statistical analysis was performed using two‐way ANOVA with Tukey's multiple comparison test. *P*‐value ** < 0.01, *** < 0.001, **** < 0.0001.

In human T1D, it has been well documented that a reduction in bacteria capable of producing short‐chain fatty acids such as butyrate is present before and after disease onset.^30^, ^31^, ^32^, ^33^ The delivery of short‐chain fatty acid yielding diets can prevent diabetes onset in NOD mice and modulate the systemic immune response in humans with T1D.^5^, ^34^ To test whether the bacterial metabolite butyrate could reduce the effector function of IGRP_206–214_ specific T cells in the PP, we administered soluble butyrate in the drinking water for 2 weeks and then performed an *in vivo* CTL assay (Figure 6). IGRP_206–214_ specific killing was again highest in the PP in the control NOD mice. Following butyrate administration, IGRP_206‐214_ specific killing was reduced in all tissues. This supports that autoreactive T cells circulating through the GALT are exposed to bacterial derived metabolites that alter their activation status.

## DISCUSSION

Current evidence suggests that an altered gut microbiota plays a role in the pathogenesis of T1D, but the exact mechanisms through which bacteria in the gut may influence the activation of islet‐specific T cells is unclear.^13^ Here we have investigated whether two major specificities of islet‐specific CD8^+^ T cells are activated either directly or indirectly within the GALT. We show that both IGRP_206–214_ and InsB_15–23_ specific CD8^+^ T cells undergo primary activation within the PcLN in the presence of endogenously derived antigen but not in the MLN or PP, arguing against a role for molecular mimicry in activation of these cells. Pre‐activated, effector‐memory phenotype islet‐specific CD8^+^ T cells circulate into the PP and these cells have increased cytotoxic function and expression of cytotoxic granzyme B compared with other sites. The microbially produced metabolite butyrate can suppress cytotoxic function by islet‐specific CD8^+^ T cells. These data support that islet‐specific T‐cell activation can be modulated by the gut microbiota independently from the presence of cross‐reactive peptide epitopes derived from the microbiota.

Numerous studies have shown that there is an altered composition and function of the gut microbiota prior to disease onset in both humans and mice at increased risk of developing autoimmune diabetes.^5^, ^11^, ^30^, ^32^, ^35^, ^36^ As the gut microbiota contain trillions of individual bacteria belonging to thousands of species and sub‐species, they comprise a large source of potentially antigenic peptides. Furthermore, it is now well understood that individual TCR (including self‐antigen specific TCR) can recognize many different peptide epitopes with varying affinity.^37^, ^38^ For example, a human preproinsulin‐reactive, MHC class‐I restricted CD8^+^ T‐cell clone (1E6) was found to recognize over a million different peptides.^21^ Several studies have identified the presence of epitopes within the gut microbiota peptidome that are cross‐reactive with known islet antigens of relevance to T1D progression.^6^, ^10^, ^20^, ^23^ However, the high inter‐individual variability in the gut microbiota and different population groups plus the highly variable relative abundance of individual species means that it is hard to know whether the presence of such peptide mimics will impact the activation of islet‐specific T cells *in vivo*. Our data suggest that commensal bacteria‐derived peptide antigens able to productively activate IGRP_206–214_ and InsB_15–23_ specific CD8^+^ T cells were either not present in the gut microbiota of the NOD or B16A‐NOD mice or were not transported to the GALT for antigen presentation to T cells, perhaps due to exclusion by the mucous barrier.

While we did not find evidence of molecular mimicry leading to activation of IGRP_206–214_ and InsB_15–23_ specific CD8^+^ T cells in our mice, we did observe preferential migration of effector‐memory 8.3 and G9C8 T cells into the PP. The IGRP‐specific T cells found within the PP had increased expression of granzyme B and cytotoxic capacity compared with the other sites we tested, suggesting that they may become further activated by gut‐derived inflammatory signals within the PP. The islet‐specific T cells did not accumulate in the PP as the overall numbers and proportion of cells remained low. InsB_15‐23_ specific CD8^+^ T cells are thought to be activated and are found in higher numbers at an earlier time point than we studied (6–8 weeks of age), therefore it is possible that more activated G9C8 cells would be observed in the PP in younger mice. This suggests that these cells may be further activated by gut‐derived signals while on route from the PcLN to the pancreas.

We do not know whether the microenvironment within the PP caused the islet‐specific T cells to become more activated, potentially due to inflammatory signals derived from the gut microbiota, or whether more activated cells preferentially migrated into the PP. Previously, it was shown that dendritic cells isolated from the PP, spleen and peripheral LN of C57BL/6 mice could all generate CTL equally in *ex vivo* assays, but PP dendritic cells imprint gut homing *via* inducing α4β7 expression.^39^ Further studies are needed to investigate the mechanism behind the increased T cell activation in the gut such as characterizing the dendritic cell phenotype in the NOD mouse.

Previous reports have shown that islet‐specific T cells can travel from the gastrointestinal tract and peritoneum to the PcLN and islets.^4^, ^40^ This process was enhanced by induction of damage to the intestinal epithelium *via* low‐dose dextran‐sulfate sodium treatment.^4^ Furthermore, islet‐specific T cells within the PcLN express the mucosal homing marker α4β7 integrin.^4^ We now show that the reverse process also occurs, with migration of islet‐specific cells from the PcLN to the GALT. This finding is in line with a previous report showing aberrant re‐circulation of α4β7^+^ T cells in NOD mice, which migrated to non‐mucosal as well as mucosal sites compared with non‐diabetes prone strains.^41^ Furthermore, others have demonstrated that MLN and PcLN derived lymphocytes but not lymphocytes from other lymph nodes can transfer diabetes into NOD.SCID recipients.^42^ This suggests a two‐way trafficking between the PcLN and gut tissues may occur in NOD mice.

Importantly, the outcome of islet‐specific T‐cell activation within the GALT may vary depending on the functional properties of the gut microbiota present. To test this, we administered oral butyrate to our mice to increase the availability of this tolerogenic metabolite known to be reduced in both NOD mice and human T1D.^5^, ^30^, ^31^, ^33^ Butyrate is produced by the gut microbiota during fermentation of dietary fiber and can delay diabetes in NOD mice.^5^, ^43^ The reduction in IGRP‐specific cytotoxicity in the presence of oral butyrate, including within the PP, confirmed that a gut microbiota produced metabolite can modulate the effector function of islet‐specific CD8^+^ T cells. Although we do not know whether this reduction in cytotoxicity was due to a direct effect of butyrate on the CD8^+^ T cells, or an indirect effect *via* expansion of regulatory T cells as reported previously.^5^ Interestingly the effect of butyrate was systemic, perhaps indicating that a high dose of butyrate led to increased circulating plasma butyrate. While we did not examine the activation status or cytotoxicity of the islet‐infiltrating T cells, it is possible that butyrate or the gut microbiota also impacted these cells and this requires further investigation.

Our data suggest that the influence of the gut microbiota on islet‐specific autoimmunity may be as an amplifying (or suppressive) factor rather than a disease triggering event. This is in line with several pieces of evidence from human studies. Kostic *et al*. showed that an alteration in the gut microbiota of children at risk of T1D was most evident in the period after the onset of islet autoimmunity but prior to disease progression, suggesting the microbiota may accelerate disease progression.^44^ Epidemiological evidence shows that while the rate of T1D progression has been rising over time, the incidence of islet autoimmunity has not changed.^45^ This suggests that environmental drivers may be accelerating the speed of progression to T1D rather than the events that trigger islet autoimmunity.

While we have shown that molecular mimicry was not responsible for driving the activation of IGRP_206‐214_ and InsB_15‐23_ specific CD8^+^ T cells in our NOD mice, this does not exclude the possibility that bacteria‐derived epitope mimics may be present in other mouse colonies or exist for other islet‐specific T cell epitopes and in relevant human gut microbial communities. We also did not perform metagenomic sequencing to profile the microbiome of our mice and to confirm whether any mimic peptides were predicted to be present. However, our study shows that irrespective of the presence of microbiota‐derived peptide antigens, the GALT environment still influences the activation status of islet‐specific T cells.

In conclusion, we demonstrate a link between the gut microenvironment and gut‐associated lymphoid tissues and effector function of islet‐specific T cells. This inter‐relationship is not dependent on the presence of cross‐reactive peptide epitopes derived from the gut bacteria to drive activation of islet‐specific T cell clones. Rather, it shows that gut‐bacteria‐derived factors can have a broad impact on autoreactive T cells, including the driving of tolerance induction.

## METHODS

### Animals

Female NOD/ShiLtJArc were purchased from the Animal Resources Centre (Perth, WA, Australia). Transgenic G9C8 NOD mice express an InsB_15‐23_ specific TCR and produce InsB_15‐23_ specific CD8^+^ T cells.^46^ CD90.1^+^ transgenic 8.3 NOD mice^47^, ^48^, ^49^ express IGRP_206–214_ specific TCR and produce IGRP_206–214_ specific CD8^+^ T cells, were bred in the TRI Biological Resources Facility. CD90.1 is a congenic marker allele, previously back‐crossed from onto the NOD background.^50^ Female B16A mice expressing a mutated pre‐proinsulin on the 16th amino acid of the insulin B chain together with double knockout native *Ins1* and *Ins2* genes^14^ were bred at the Translational Research Institute Biological Research Facility (Brisbane, QLD, Australia). All mice used were housed in specific pathogen‐free conditions. Female animals used in all experiments were aged between 12 and 16 weeks and were non‐diabetic. All experimental procedures were approved by the University of Queensland Animal Ethics Committee.

### Adoptive transfer of islet‐specific transgenic T cells

CD8^+^ T cells from the NOD 8.3 CD90.1^+^ mice or G9C8 mice were isolated from pooled spleen and lymph node cells. Transgenic 8.3 and G9C8 cells were mixed at a 1:1 ratio and then labeled with 5 μM CellTrace Violet (CTV, Invitrogen) in pre‐warmed PBS and incubated for 15 min at 37°C. The reaction was terminated with ice‐cold complete RPMI, washed and re‐suspended in sterile PBS for adoptive transfer. NOD/Lt animals were intravenously transferred with a mixture of 5 × 10^6^ 8.3 cells and 5 × 10^6^ G9C8 cells *via* tail‐vein injection and tissues harvested for analysis 3 days later. The transferred 8.3 T cells were identified as CD8^+^, CTV^+^, CD90.1^+^ and G9C8 cells were CD8^+^, CTV^+^, CD90.1^−^. In some experiments, naïve CD8^+^CD44^−^CD62L^+^ 8.3 cells were sorted prior to transfer.

### Oral butyrate and *in vivo* CTL assay

NOD/Lt mice (10–11 weeks old) received sodium butyrate (Sigma Aldrich, St Louis, MI, USA) in their drinking water (200 mM) for 2 weeks. The NOD mice (12–13 weeks old) were injected with a 1:1 mixture 3 × 10^6^ CTV^high^ labeled target cells and 3 × 10^6^ CTV^low^ control cells. Targets were splenocytes from 6‐week‐old NOD donor mice pulsed with IGRP_206–214_ peptide and control cells were unpulsed NOD splenocytes. The CTV^high^ cells were labeled with 5 μM CTV and CTV^low^ cells were labeled with 0.5 μM CTV. Specific killing was assessed by flow cytometry 16 h later using the formula 100 − ([CTV^high^ target %/CTV^low^ control cell % in sample] / [CTV^high^ targets %/CTV^low^ control cell % in pre‐injection sample] × 100).

### Flow cytometry

Harvested spleens, inguinal, pancreatic and mesenteric lymph nodes (ILN, PcLN, MLN) and Peyer's patches (PP) were passed through a 70‐micron cell strainer with FACS buffer containing 2% BSA “BovoStar” (Bovogen Biologicals, Keilor East, VIC, Australia) and 2 mM EDTA in 1x PBS. For MLN and PP, FACS buffer also contained 10 mg mL^−1^ DNase I (Roche, Basel, Switzerland). H‐2K^d^ IGRP_206‐214_ (VYLKTNVFL) monomers were provided by the NIH Tetramer Core Facility (Atlanta, GA, USA) and were tetramerized in accordance with the NIH Tetramer Core Facility protocol using Streptavidin‐PE (ProZyme, Hayward, CA, USA). The tetramer staining was performed in the dark at room temperature for 20 min prior to restimulation or staining. The cells were stained for surface and/or intracellular markers in the dark at 4°C using antibodies targeting CD8alpha (53–6.7, Biolegend, San Diego, CA, USA), CD44 (IM7, Biolegend), CD62L (MEL14, eBioscience, San Diego, CA, USA), CD69 (H1.2F3, Biolegend), CD90.1 (Thy1.1) (OX‐7, Biolegend), CD279 (PD‐1) (29F.1A12, Biolegend), granzyme B (GB11, Biolegend). Live/Dead FVS575V (BD Biosciences, Franklin Lakes, NJ, USA) was used to discriminate between live and dead populations. Intracellular staining was performed post‐extracellular staining, with cells fixed and permeabilized using 200 μL Cytofix/CytoPerm reagents (BD Biosciences, Franklin Lakes, NJ, USA). Flow cytometry was performed using a LSR Fortessa X‐20 (BD Biosciences).

### Statistical analyses

Samples with < 20 tetramer+ events or 8.3 or G9C8 cells events were excluded from phenotyping analysis. One‐way, two‐way ANOVA or repeated measures ANOVA were used to compare groups with Tukey's comparison for post‐hoc analysis as appropriate using GraphPad Prism Version 8 (Graphpad, San Diego, CA, USA). Significance was determined as *P ≤ 0.05, **P ≤ 0.01, ***P ≤ 0.001, ****P ≤ 0.0001.

## AUTHOR CONTRIBUTIONS

**Mirei Okada:** Formal analysis; investigation; visualization; writing – review and editing. **Vivian Zhang:** Formal analysis; investigation; writing – review and editing. **Jeniffer Denisse Loaiza Naranjo:** Formal analysis; visualization; writing – review and editing. **Bree Tillett:** Conceptualization; supervision; writing – review and editing. **Susan Wong:** Resources; writing – review and editing. **Raymond Steptoe:** Resources; writing – review and editing. **Anne‐Sophie Bergot:** Conceptualization; supervision; writing – review and editing. **Emma Estelle Hamilton‐Williams:** Conceptualization; formal analysis; funding acquisition; project administration; resources; supervision; writing – original draft.

## CONFLICTS OF INTEREST

The authors have no conflicts of interest to declare.

## Data Availability

Data are available from the authors on request.
