# Supplementary material for: Islet‐specific CD8 + T cells gain effector function in the gut lymphoid tissues via bystander activation not molecular mimicry
Source: Immunol Cell Biol. 2022 Nov 1;101(1):36–48. doi: 10.1111/imcb.12593 (PMC10092732; doi:10.1111/imcb.12593)
Supplement: Supplementary file 1 [file IMCB-101-36-s001.pdf]

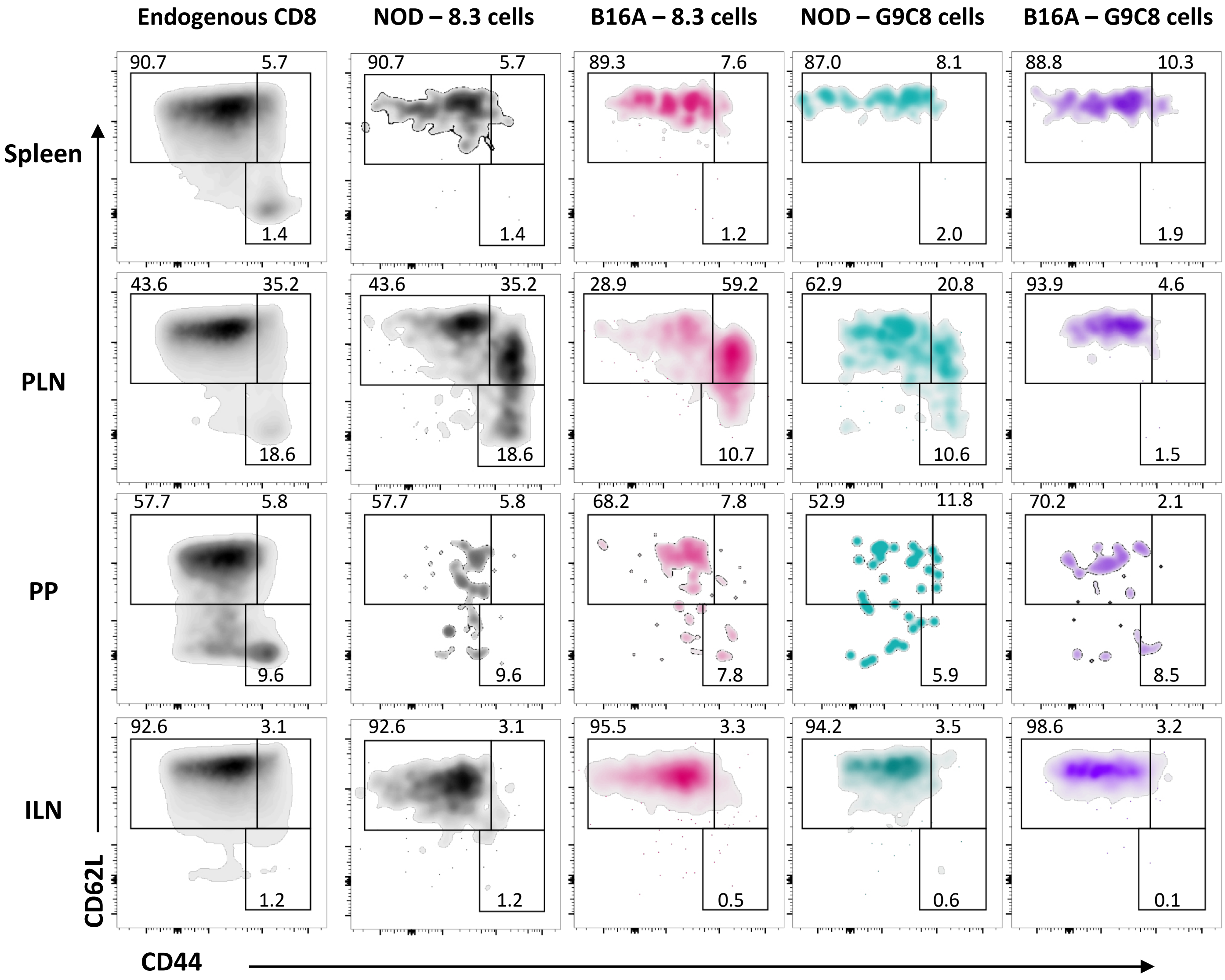

Supplementary figure 1. Representative CD44 and CD62L staining on 8.3 and G9C8 cells. Cells were stained with CD44 and CD62L. Examples from spleen, PcLN, PP, ILN and endogenous CD8 cells. 8.3 cells from NOD and B16A recipient mice; G9C8 cells from NOD and B16A recipient mice.
